# Phylogenomic Relationships of Diploids and the Origins of Allotetraploids in *Dactylorhiza* (Orchidaceae)

**DOI:** 10.1093/sysbio/syz035

**Published:** 2019-05-25

**Authors:** Marie K Brandrud, Juliane Baar, Maria T Lorenzo, Alexander Athanasiadis, Richard M Bateman, Mark W Chase, Mikael Hedrén, Ovidiu Paun

**Affiliations:** 1 Department of Botany and Biodiversity Research, University of Vienna, Rennweg 14, A-1030 Vienna, Austria; 2 Royal Botanic Gardens Kew, Richmond, Surrey, TW9 3AB, UK; 3 Department of Environment and Agriculture, Curtin University, Bentley, Western Australia 6102, Australia; 4 Department of Biology, University of Lund, Sölvegatan 37, SE-223 62 Lund, Sweden

**Keywords:** Allopolyploidy, coalescent, *Dactylorhiza*, phylogenomics, RADseq, reticulate evolution, speciation

## Abstract

Disentangling phylogenetic relationships proves challenging for groups that have evolved recently, especially if there is ongoing reticulation. Although they are in most cases immediately isolated from diploid relatives, sets of sibling allopolyploids often hybridize with each other, thereby increasing the complexity of an already challenging situation. *Dactylorhiza* (Orchidaceae: Orchidinae) is a genus much affected by allopolyploid speciation and reticulate phylogenetic relationships. Here, we use genetic variation at tens of thousands of genomic positions to unravel the convoluted evolutionary history of *Dactylorhiza*. We first investigate circumscription and relationships of diploid species in the genus using coalescent and maximum likelihood methods, and then group 16 allotetraploids by maximum affiliation to their putative parental diploids, implementing a method based on genotype likelihoods. The direction of hybrid crosses is inferred for each allotetraploid using information from maternally inherited plastid RADseq loci. Starting from age estimates of parental taxa, the relative ages of these allotetraploid entities are inferred by quantifying their genetic similarity to the diploids and numbers of private alleles compared with sibling allotetraploids. Whereas northwestern Europe is dominated by young allotetraploids of postglacial origins, comparatively older allotetraploids are distributed further south, where climatic conditions remained relatively stable during the Pleistocene glaciations. Our bioinformatics approach should prove effective for the study of other naturally occurring, nonmodel, polyploid plant complexes.

Disentangling phylogenetic relationships often proves challenging in groups that have diverged recently and/or rapidly (Mallo and Posada 2016; Fernández-Mazuecos et al. 2017). Problems encountered include minimal morphological and genetic differentiation, phenotypic convergence, widespread incomplete lineage sorting, and porous genomes subject to interspecific gene flow. Molecular phylogenetic approaches have typically proposed to investigate as many genomic regions as possible by combining them, with the general expectation that the history of the majority of genes will accurately reflect history (i.e., the “species tree”; Ebersberger et al. 2007). However, due to coalescent stochasticity along the genome, individual gene trees are likely to be incongruent with each other and with the species tree (Degnan and Rosenberg 2006; Liu et al. 2015; Pease et al. 2016). Hence, it has become clear that, although reasonably accurate when there is little heterogeneity among gene trees, concatenation methods have limitations due to issues such as long-branch attraction, heterogeneity in rates of substitutions among closely related lineages, and limited sampling of lineages. Multispecies coalescent approaches have recently been suggested as appropriate alternatives, but they are computationally intensive, sensitive to inaccurate species circumscriptions, and vulnerable to incongruence caused by interspecific gene flow (Liu et al. 2015; Mallo and Posada 2016)—the last a putatively omnipresent phenomenon in plants and animals (Taylor and Larson 2019).

Understanding evolutionary histories is further complicated by hybrid and polyploid entities that exhibit mixed ancestral alleles and inheritance (Dufresne et al. 2014; Meirmans et al. 2018). Hybridization and polyploidy are, however, pervasive evolutionary processes driving speciation and adaptation (Ramsey and Schemske 1998; Adams and Wendel 2005; Van der Peer et al. 2017; Taylor and Larson 2019). Allopolyploids integrate distinct parental genomes and are typically reproductively isolated from their parents (Soltis et al. 2014). Allopolyploid genotypes often originate recurrently, for example in *Tragopogon* (Soltis et al. 2004) and *Mimulus* (Vallejo-Marin et al. 2015). As sequentially produced allotetraploids may subsequently interbreed, genetic diversity and genome complexity of these allopolyploids is enhanced (Soltis and Soltis 1999; Soltis et al. 2014). In some cases, such recurrently produced allopolyploids establish independent species that remain genetically distinct, even in sympatry, for example in *Achillea* (Guo et al. 2013), *Asplenium* (Perrie et al. 2010), *Leucaena* (Govindarajulu et al. 2011), and *Oryza* (Zou et al. 2015).

The parental taxa of allopolyploids typically have been genetically isolated for a relatively long period (e.g., Paun et al. 2009). However, due to heterogeneity in parental divergence along the genome, gene conversion, and illegitimate recombination, most allopolyploids will exhibit mixed inheritance (Stift et al. 2008). This, together with the slow development of population genetic theory for polyploids, difficulties in clearly identifying homeologs, and allelic dosage uncertainty have impaired investigation of polyploid evolution (Blischak et al. 2016; Meirmans et al. 2018).

Methods that use likelihoods to integrate over the uncertainty of inferring genotypes have been shown to dramatically improve accuracy when analyzing intermediate- to low-coverage sequence data (Nielsen et al. 2011; Vieira et al. 2016). In addition to accounting for uncertainty in high-throughput sequencing data, models of calling biallelic single nucleotide polymorphisms (SNPs) based on likelihoods can overcome biases associated with genotype uncertainty in polyploids (Blischak et al. 2018).

In this article, we explore the utility of restriction site-associated DNA sequencing (RADseq; Baird et al. 2008) for inferring evolutionary patterns in an orchid genus (*Dactylorhiza* Necker ex Nevski) that comprises numerous allotetraploids, an autotetraploid, and their putative diploid progenitors. As a consequence of the variability of restriction sites at a broader phylogenetic scale, the proportion of homologous sequences obtained by RADseq will decrease with phylogenetic distance, which may be problematic when distantly related taxa are included in analyses. However, RADseq has been estimated to be useful for resolving divergences as old as 63 myr (Cariou et al. 2013; Heckenhauer et al. 2018), while also efficiently resolving recently radiating groups (e.g., Cruaud et al. 2014; Paun et al. 2016; Trucchi et al. 2017; Bateman et al. 2018b; Brandrud et al. 2019).

*Dactylorhiza* is a temperate-boreal genus with the main distribution in Europe and a few species in Asia, North America, and the mountains of North Africa (Averyanov 1990; Pillon et al. 2007). The genus has not previously been assessed with such a broad sampling of taxa or genomic loci. Due to morphological heterogeneity and frequent interspecific hybridization, *Dactylorhiza* is considered taxonomically controversial (Pillon et al. 2006); the number of species recognized varies between 6 and 75 [reviewed by Pedersen (1998); see also Supplementary Table S1; available on Dryad at http://dx.doi.org/10.5061/dryad.j01ph32]. Published molecular analyses, which in general included few *Dactylorhiza* accessions within a broader phylogenetic context, have employed from one to only a few molecular markers (e.g., nrITS—Bateman et al. 2003 and Pillon et al. 2007; nrITS and ETS—Devos et al. 2006; mitochondrial *cox1* intron—Inda et al. 2010; nrITS, a plastid *rpl16* intron and a mitochondrial *cox1* intron—Inda et al. 2012) and generally resulted in poor resolution and often conflicting topologies (Bateman et al. 2018a). Even greater taxonomic controversy has afflicted concepts of the numerous *Dactylorhiza* allotetraploids, which predominantly originated from hybridization between two broadly defined parental groups, the *D. fuchsii-maculata* and the *D. incarnata-euxina* clades (Heslop-Harrison 1953; Hedrén 1996, 2001; Pedersen 2004; Hedrén et al. 2007). However, due to the high frequencies of extensive reticulation within the *Dactylorhiza* allotetraploid complex, previous efforts to resolve their origins (e.g., Pillon et al. 2007) have yielded ambiguous results.

We use here a comprehensive approach to obtain detailed insights into diploid and polyploid evolution within this complex genus and assess the utility of RADseq for studying reticulate evolution in polyploid complexes. The current study first employs thousands of SNPs with coalescent methods to delimit diploid *Dactylorhiza* species and estimate their phylogenetic relationships. The delimited diploid species and a synthetic reference based on diploids only are then employed with ploidy-aware, genotype likelihood-based methods of estimating allele dosage to elucidate the origins of allotetraploids within the genus, including determining the direction of the hybridization events that produced them. The analytical approach taken here is powerful in disentangling convoluted evolutionary relationships and should prove effective for application to other polyploid complexes.

## Materials and Methods

### Plant Material

**Figure 1.**
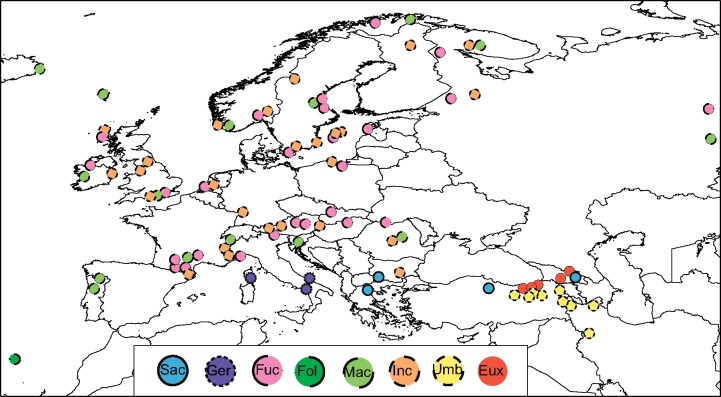
Map showing the sampling locations of 85 diploid and 18 autotetraploid *Dactylorhiza* individuals analyzed here as representatives of potential parents of allopolyploids. The study further includes nine diploid individuals of *D. aristata, D. iberica, D. sambucina*, and *D. viridis*, for which sampling locations are presented exclusively in Supplementary Table S1 available on Dryad. Eux = *D. euxina*, Fol = *D. foliosa*, Fuc = *D. fuchsii*, Inc = *D. incarnata*, Mac = *D. maculata*, Sac = *D. saccifera*, Umb = *D. umbrosa*. The map layer was extracted from GADM version 1.0 (available from www.gadm.org). Exact coordinates are given in Supplementary Table S2 available on Dryad.

**Figure 2.**
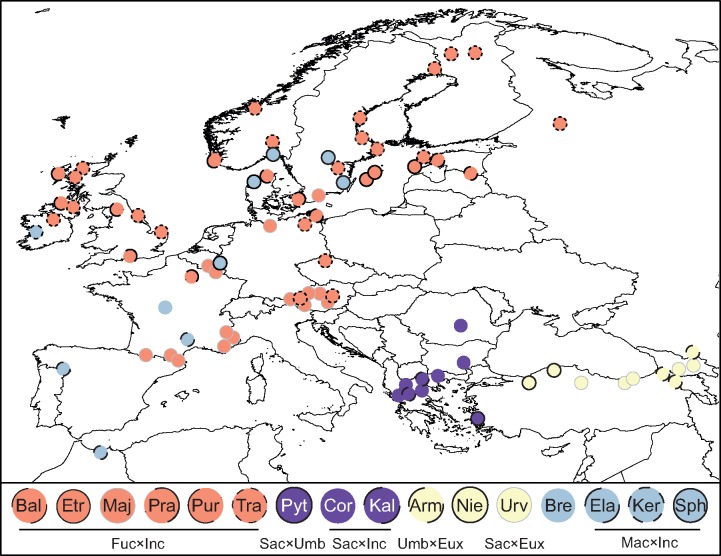
Maps showing the sampling locations of 95 allotetraploid *Dactylorhiza* accessions studied. Arm = *D. armeniaca,* Bal = *D. baltica* (incl. *D. ruthei*), Bre = *D. brennensis,* Cor = *D. cordigera*, Ela = *D. elata*, Etr = *D. elatior*, Kal = *D. kalopissii* (incl. *D. macedonica*), Ker = *D. kerryensis*, Nie = *D. nieschalkiorum*, Maj = *D. majalis*, Pra = *D. praetermissa*, Pur = *D. purpurella*, Pyt = *D. pythagorae*, Sph = *D. sphagnicola* (incl. *D. calcifugiens*), Tra = *D. traunsteineri,* Urv = *D. urvilleana*. Lineages involved in the origin of the allopolyploids are also indicated: Eux = *D. euxina*, Fuc = *D. fuchsii*, Inc = *D. incarnata*, Mac = *D. maculata*, Sac = *D. saccifera*, Umb = *D. umbrosa*. The map layer was extracted from GADM v. 1.0 (available from www.gadm.org). Exact coordinates are given in Supplementary Table S2 available on Dryad.

Our sampling covers most diploids in the genus (apart from a few poorly documented diploids in the Himalayas and China) and 16 allotetraploid entities. Altogether, we included 94 diploid, 18 autotetraploid, and 95 allotetraploid accessions of *Dactylorhiza* (Figs. 1 and 2, Supplementary Table S1 available on Dryad). When possible, we included several accessions of each putative *Dactylorhiza* species, although for the diploid *D. aristata* and two allotetraploids only single individuals were available. Additionally, 18 diploids from related genera were sampled. Vouchers have been deposited in the herbaria of Lund University (LD; Supplementary Table S2 available on Dryad). Maps of the sampling locations for the parental species and allotetraploids were generated using QGIS v. 2.4.071 (QGIS Development Team 2018), constructed on a map layer extracted from GADM v. 1.0 (available from www.gadm.org).

Depending on their degree of morphological, ecological, reproductive, and molecular distinctiveness, and on the taxonomic criteria applied, *Dactylorhiza* allotetraploids have in the past been variously treated as species, subspecies, varieties, formae, or various combinations of these ranks (Supplementary Table S1 available on Dryad). In the following text, for simplicity and neutrality, all polyploids are referred to with a Latin epithet but without assignment to a particular taxonomic rank.

### DNA Extraction, Library Preparation, and Sequencing

Total DNA was isolated from silica-dried leaves or flowers/bracts using a cetyl trimethylammonium bromide (CTAB) procedure (Doyle 1990) or the DNeasy Plant Mini Kit (Qiagen, Venlo, Netherlands). DNA was purified with the Nucleospin gDNA clean-up kit (Macherey-Nagel, Düren, Germany) following the manufacturer’s protocol. RADseq libraries of 30–72 individuals per library, including repeats of individuals as necessary, were prepared following the protocol of Paun et al. (2016) with the following modifications. Depending on the library, for each sample 100–400 ng DNA was used, except for the repeated individuals where only 50–100 ng was used. Aiming for equal coverage per allele across ploidies, only half the number of individuals per library and twice the amount of DNA was used for tetraploid accessions in comparison with diploids. The DNA content of samples was normalized at the level of each library. DNA was sheared with a Bioruptor Pico using 0.65 mL tubes (Diagenode) and three cycles of 30 s ON and 60 s OFF. The inline and index barcodes used differed from each other by at least three sequence positions. All RADseq libraries were sequenced as single-end 100 bp reads on an Illumina HiSeq platform at VBCF NGS Unit (https://www.vbcf.ac.at/ngs), Vienna, Austria.

In addition, one individual of *D. fuchsii* from Austria (accession 2144) was used to construct a whole genome sequencing (WGS) library using a TruSeq DNA PCR-Free Library Kit (Illumina Inc.) according to manufacturer’s instructions. This was sequenced as a spike-in pair-end 125 bp reads on an Illumina HiSeq at VBCF NGS Unit and used to build a reference plastid genome with Fast-Plast v. 1.2.6 (available at: https://github.com/mrmckain/Fast-Plast) employing Asparagales in the –bowtie_index option. Plastome annotations were performed online using GeSeq (Tillich et al. 2017), guided with NCBI reference annotations for *Habenaria pantlingiana* and *H. radiata* (species belonging to the same orchid subfamily as *Dactylorhiza*—Orchidoideae).

### Filtering SNPs from RADseq Data

To allow for phylogenomic investigations at the genus level across nonmodel diploids and polyploids (i.e., in the absence of a reference genome), we optimized a bioinformatics pipeline by building a synthetic reference from all diploid accessions, later mapping both diploids and polyploids to this reference. Finally, we called and filtered variants across all samples by taking into account the ploidy of each accession.

The raw reads were first demultiplexed to sublibraries based on index reads using BamIndexDecoder v. 1.03 (included in Picard Illumina2Bam package, available from http://gq1.github.io/illumina2bam/). These were further processed with STACKS v. 1.44 (Catchen et al. 2013), starting with demultiplexing of individuals based on inline barcodes via PROCESS_RADTAGS. Simultaneous quality filtering was performed with default options, rescuing barcodes, and cut sites with a maximum of one mismatch relative to expectation. RADseq loci were initially built *de novo* for the set of diploid individuals with DENOVO_MAP.PL in STACKS. Following Paun et al. (2016), we first varied the settings for catalog building, and we optimized them to maximize the likelihood of ortholog recovery across species while avoiding collapse of paralogs (i.e., the criterion used was to maximize the number of polymorphic loci that contain a maximum of ten SNPs present in at least 90% of individuals). The final settings chosen required at least six reads to create a stack (m), allowing for a maximum of one mismatch when merging the loci within individuals (M) and also among individuals when building the catalog (n). The setting allowing for indels was tested, but it did not improve significantly the number of loci recovered and was abandoned. Finally, we retained all polymorphic RADtags covered in at least 50% of individuals that do not have more than 15 SNPs with EXPORT_SQL.PL in STACKS. A consensus for each locus has been retained as individual contigs to produce a FASTA reference for further analysis.

In the next step, the raw reads of diploids and polyploids were mapped back to this diploid-derived reference using BOWTIE2 v. 2.2.6 (Langmead and Salzberg 2012) with default settings. The SAM files have been further converted to BAM, sorted by reference coordinates and read groups were added with Picard v. 2.6.0 (available from http://broadinstitute.github.io/picard). Realignments around indels have been performed using the Genome Analysis Toolkit v. 3.8 (GATK; McKenna et al. 2010). Two approaches have been used for further analyses. First, genotypes were called with REF_MAP.PL and POPULATIONS in STACKS using default settings for diploids and autotetraploids for phylogenetic analyses. VCFTOOLS v. 0.1.14 (Danecek et al. 2011) was then used to filter this data set to retain biallelic SNPs covered in at least 75%, 90%, or 95% of individuals, depending on the downstream analysis (see below and Supplementary Table S3 available on Dryad). For most analyses all SNPs have been included, whereas for coalescent-based analyses only one SNP per RADtag was retained. The filtered vcf file was converted to other formats with PGDSpider v. 2.0.8.2 (Lischer and Excoffier 2011).

Second, for all individuals including the allotetraploids we used the realigned BAM files and the GATK UnifiedGenotyper to call variants and estimate genotypes. This was done separately for diploids (with -ploidy 2) and tetraploids (with -ploidy 4). We then followed an approach proposed by Blischak et al. (2018) to refine the genotypes based on normalized genotype likelihoods with flat priors (i.e., all genotypes are assumed to be equally likely). Although this approach ignores the fact that genotypes are drawn from two independent subgenomes in allopolyploids, it takes into account the ploidy of each accession, and for high levels of sequence coverage (as for our data; see below) it has negligible levels of estimation errors (Blischak et al. 2018). In addition, as a major aim of our study was to identify the parents of allotetraploids, we could not use more sophisticated approaches of calling allopolyploid genotypes based on prior knowledge of allele frequencies in parental populations.

In short, we used the filter-vcf.R and intersect-vcf.R scripts provided with EBG v. 0.3.2-alpha (Empirical Bayes Genotyping in Polyploids; Blischak et al. 2018) to retain only biallelic SNPs with data for at least 50% of individuals, and which had a minimum quality threshold of 100, a minimum read depth of five, and were shared between both the diploid and tetraploid variant files. The resulting VCF files were used to extract base quality scores from the original BAM files using the mpileup command of SAMtools v.1.6 (Li 2011) and to estimate an error rate for each variant position with the per-locus-err.py script provided with EBG. Finally, the genotypes were inferred as derived allele counts with EBG gatk and 10,000 iterations, taking into account the total read count, the derived read count, and the error rate at each position.

Finally, the demultiplexed RADseq reads were also mapped to the *de novo* assembled plastid reference genome of *D. fuchsii* with the MEM algorithm of BWA v. 0.7.12-r1039 (Li and Durbin 2009). After sorting the aligned SAM files by coordinates, adding read groups with Picard tools v. 2.9.2 (available from http://broadinstitute.github.io/picard), and realigning around indels with the GATK, variants were then called using the GATK HaplotypeCaller in the ERC GVCF mode with a sample ploidy of one. Finally, joint genotyping was performed on the resulting gVCF files with the GenotypeGVCFs tool of GATK.

### Diploid Phylogenomic Analyses

Based on REF_MAP.PL-based, filtered SNP data set for diploid and autotetraploid individuals (i.e., *D. maculata*), we constructed phylogenetic trees with RAxML v. 8.2.9 (Stamatakis 2014). We used an algorithm with 1000 rapid bootstrap replicates while searching for the best-scoring ML tree under the general time reversible (GTR) model of nucleotide substitutions with disabled rate heterogeneity among sites (i.e., the GTRCAT model). As recommended in the RAxML v. 8.2.X manual, we also applied an ascertainment bias correction of the likelihood following the method proposed by Lewis (2001) for data sets of concatenated SNPs, with 1000 alternative runs on distinct starting trees. We aimed to investigate further a potential effect of gene flow between *D. fuchsii* and autotetraploid *D. maculata* in Central Europe (Ståhlberg and Hedrén 2009, 2010) on the recovered topology; hence, RAxML analyses have also been performed with the same settings on a matrix that included diploids plus only the western European autotetraploid $D$. *maculata.* The trees were visualized with FIGTREE v. 1.4.2 (available from http://tree.bio.ed.ac.uk/software/figtree/).

For two major clades in the RAxML phylogenetic tree that contain putative parents of allotetraploids, we independently performed Bayesian species delimitation analyses (Leaché et al. 2014). The analyses were run separately in order to decrease computational time. These two analyses focused on (i) the *D. fuchsii-maculata* clade containing $D$. *fuchsii*, *D. saccifera*, *D. gervasiana*, and *D. foliosa* (plus *D. incarnata* as their sister) and (ii) the *D. incarnata-euxina* clade containing *D. incarnata*, *D. umbrosa*, “*D. osmanica*,” and *D. euxina* (plus *D. fuchsii* as their sister). For this purpose, two smaller data sets were created by including only a single biallelic SNP per RAD locus, allowing for a maximum of 10% missing data per locus. To further minimize computational time, we included in these analyses only a few representative individuals but ensured that at least three individuals per smallest tested group were present (Supplementary Table S2 available on Dryad). The vcf files were converted with PGDSpider to NEXUS format, from which XML files were created with BEAUti v. 2.4.5 (Bouckaert et al. 2014) and edited to a path-sampling analysis. Bayesian species delimitation analyses were performed in SNAPP v. 1.2.5 (Bryant et al. 2012) using 12 initialization steps and 1 million chain-lengths for each model, with data stored every 1000 generation excluding 10% of them as burn in. The coalescent rate, together with the forward ($u)$ and backward ($v)$ mutation rates were sampled from within the MCMC. Following recommendations (Bryant et al. 2012; Drummond and Bouckaert 2015, p. 124), we assumed a default option for a pure birth (Yule) model, governed by a single hyperparameter$\lambda$ representing the birth rate for the species tree, which was allowed to vary and was sampled during the MCMC. Finally, a log-likelihood correction for calculating likelihood values was used for Bayes factor test of the different models of species assignments. All other priors were left at default.

To build a species tree, SNAPP analyses further included all diploid *Dactylorhiza* species (as defined with the species delimitation analyses above) with available data for at least two individuals (i.e., all but *D. aristata*), based on a data set including single SNPs for each RAD tag with a chain-length of 20 million, saving a tree every 1000th generation. The rest of the parameters were similar to the species delimitation analyses above. Convergence of the SNAPP analysis was evaluated from the log-file with TRACER v. 1.6 (Rambaut et al. 2018). We summarized the trees from SNAPP and calculated posterior probabilities of each clade with TREEANNOTATOR v. 1.8.3. After removing the first 10% of trees as burn in, the SNAPP trees were visualized as a cloudogram using DENSITREE v. 2.2.6 (Bouckaert and Heled 2014). To infer ages for divergence events, the species tree was calibrated with a general mutation rate from *Arabidopsis* (7 $\times$ 10$^{-9}$ base substitutions/site/generation; Ossowski et al. 2010) and an estimated average generation time for *Dactylorhiza* of 5.8 years (Øien and Moen 2002). A similar mutation rate was recorded for *Oryza* (7.1 $\times$ 10$^{-9}$ base substitutions/site/generation; Lynch et al. 2016). The results were rescaled according to the total length of investigated sites within the included loci and the total number of polymorphic sites across this length.

To further explore relationships between diploid *Dactylorhiza* accessions and autotetraploid *D. maculata*, we calculated a pairwise relatedness matrix based on a method-of-moment with POLYRELATEDNESS v. 1.8 (Huang et al. 2014). The patterns were visualized as a heatmap, produced with the heatmap.2 function from the package GPLOTS v. 3.0.1. (available from https://cran.r-project.org/package=gplots) in R. Finally, for each diploid individual, a relative measure of inbreeding $F$, derived from a method of moment, was calculated with VCFTOOLS (–het option) and visualized for each species as violin plots with R v. 3.2.3 in RSTUDIO v. 1.0.44 (RStudio Team 2015). Resembling the classic population genetic measure $F_{IS}$, this per-individual estimate of $F$ can in theory span values from –1 (maximum outcrossing) to +1 (maximum inbreeding), but should be regarded only as a relative measure of inbreeding, as it is based on the heterozygosity of variable loci within the data set (i.e., it derives from a vcf file) and is not calculated over populations.

### Allotetraploid Analyses

To assess relationships between allotetraploids, starting from an EBG-derived variant file with heterozygous positions encoded as IUPAC codes, a phenetic network for all allotetraploid accessions was constructed with SPLITSTREE4 (Huson and Bryant 2005) using Jukes Cantor distances. As an alternative, a heatmap of coancestry between individuals was constructed with the heatmap.2 function of GPLOTS in R. The heatmap was drawn based on the relatedness coefficient proposed by Ritland 1996, as implemented in POLYRELATEDNESS (Huang et al. 2015) starting from EBG-derived genotypes of the allotetraploid individuals.

To track the parentage of each tetraploid entity, we estimated the Ritland (1996) pairwise relatedness between individuals based on the EBG-called genotypes. Relatedness coefficients, in contrast with distance metrics, do not present any bias with respect to ploidy and can therefore be used on data sets with mixed ploidy (Meirmans et al. 2018). In particular, Ritland’s coefficient of ancestry implements a maximum likelihood method that can be used to obtain relatedness between individuals of different ploidies, after correcting the estimators by multiplication of ploidy to convert to relatedness coefficients (Huang et al. 2015). Here, we inferred the diploid or autotetraploid (in the case of *D. maculata*) accessions with the highest relatedness to an allotetraploid to be the most likely parents. The distribution of relatedness between each tetraploid group and each of the potential parental taxa was presented as violin plots in R. To test the significance of the observed difference between overlapping relatedness distributions, statistical tests were performed with Mann–Whitney–Wilcoxon tests in R (command wilcox.test()) because distributions were not necessarily normally distributed. To determine from which parent each allotetraploid received its plastid genome (i.e., to identify the maternal parent), a relatedness estimator between the allotetraploids and their putative parents was calculated with a method-of-moments (Huang et al. 2014) as implemented in POLYRELATEDNESS based on the plastid-derived VCF file. The diploid with the highest plastid similarity was inferred to be the maternal parent.

Finally, as an estimate of relative age of each allotetraploid entity, the number of private alleles per allotetraploid compared with its sibling lineages was calculated based on EBG-derived genotypes. The estimates of private alleles were finally corrected for small sample sizes by multiplication with a factor (($n$ + 1)/$n)$, where $n$ represents the sample size of the relevant group.

## Results

After demultiplexing and filtering the raw reads, our RADseq data averaged 1.6 million (SD 1.3 million) high-quality reads per diploid individual and 1.8 million (SD 1.0 million) per tetraploid individual. These data have been deposited in the NCBI Short Reads Archive (BioProject ID PRJNA489792, SRA Study ID SUB4486615). The WGS raw data contained 5.4 million pair-end reads and these have been also deposited in the NCBI Short Reads Archive (BioProject PRJNA526233, experiment SRX5495602).

After parameter optimization in the *de novo* assembly pipeline of STACKS and filtering as described above, we retained 2696 polymorphic loci of 94 bp each in the “diploid” reference that was further used for mapping. On average, the diploid and tetraploid reads had a mapping success to this reference of 21.5% and 23% respectively, to a final average coverage of 194$\times$ and 215$\times$, respectively. The raw SNP data set obtained from REF_MAP.PL for the diploid and autotetraploid individuals allowing for 25% missing data contained 20,713 SNPs and no indels (Supplementary Table S3 available on Dryad). After filtering, the estimation approach implemented in EBG yielded information retained on 29,953 variable sites across the *Dactylorhiza* allotetraploids and their putative parents.

The size of the *de novo* assembled plastid genome for *D. fuchsii* is 154,007 bp (GenBank Accession number MK908418). On average, 6354 RADseq reads per accession mapped to the plastome. After variant calling and filtering to retain only variants covered in at least 50% of individuals, 767 SNPs were retained across the plastid data set that included the allotetraploids and their potential diploid parents.

### Diploid Phylogenomic Analyses

**Figure 3.**
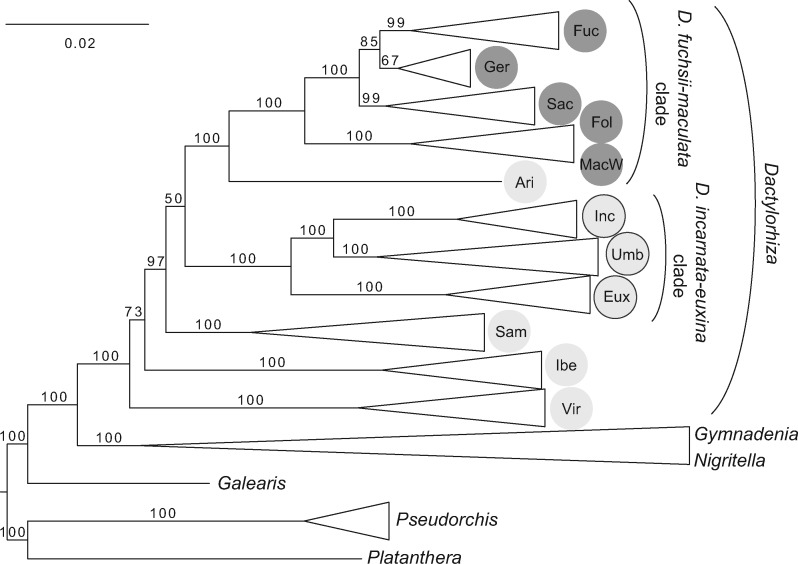
Best-scoring maximum likelihood phylogenetic tree of a data set of 20,713 SNPs, comprising 89 diploid *Dactylorhiza* individuals. Nine autotetraploid *D. maculata* accessions (MacW) from the western part of its distribution and 18 outgroup individuals from closely related genera are also included. Ari = *D. aristata*, Eux = *D. euxina*, Fol = *D. foliosa*, Fuc = *D. fuchsii*, Ibe = *D. iberica*, Inc = *D. incarnata*, Ger = *D. gervasiana*, Sac = *D. saccifera*, Sam = *D. sambucina*, Umb = *D. umbrosa* (including “*D. osmanica*” accessions), Vir = *D. viridis*.

The RAxML phylogenetic analyses performed on Eurasian *Dactylorhiza* diploids and the autotetraploid *D. maculata* (Fig. 3 and Supplementary Fig. S1 available on Dryad) distinguishes nine well-supported terminal *Dactylorhiza* groups, together with one more poorly supported represented by *D. gervasiana* individuals (bootstrap percentage, BP, 67). The single analyzed individual of *D. aristata* occupies a separate branch. With respect to the outgroup, *Dactylorhiza* is unquestionably monophyletic. The *D. fuchsii-maculata* clade, including the diploids *D. fuchsii*, *D. saccifera*, *D. gervasiana*, and *D. foliosa*, was highly supported (BP 100). When only the accessions of *D. maculata* from W Europe are included in the analysis (i.e., excluding *D. maculata* from other putative parts of the its distribution), *D. fuchsii* is sister to *D. gervasiana* comprising individuals from France and Italy (Fig. 3). When all *D. maculata* accessions are included in the RAxML analysis (Supplementary Fig. S1 available on Dryad), *D. fuchsii* is sister to *D. maculata/D. foliosa* clade, but this relationship has very low support (BP 53). The *D. incarnata-euxina* clade (including *D. umbrosa*) is well supported (Fig. 3); it is sister to the *D. fuchsii-maculata* clade but with low support (BP 50). The accessions of *D. umbrosa* and “*D. osmanica*” were placed within the same clade and intermixed. Finally, the relationships of *D. iberica* and *D. viridis* to each other and to the clade of all other *Dactylorhiza* species are unclear (BP 73; Fig. 3).

**Figure 4.**
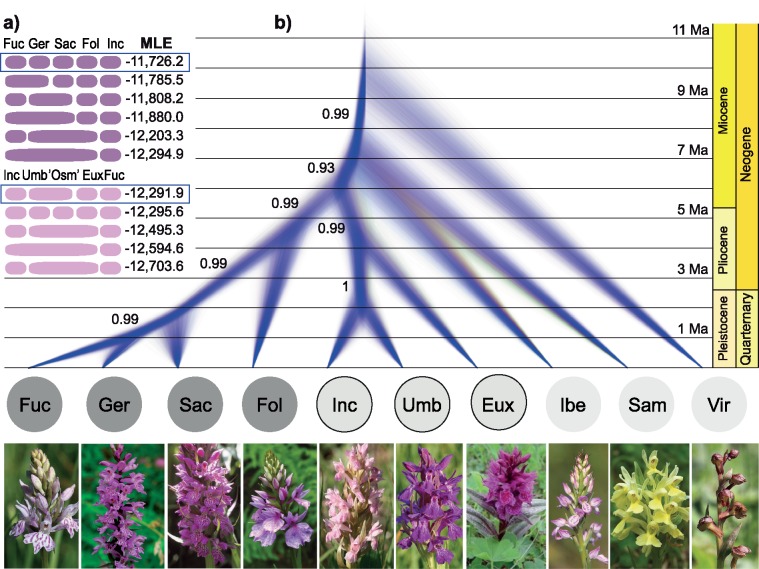
Results of coalescent-based phylogenetic analyses from SNAPP for diploid *Dactylorhiza*. a) Species delimitation models tested for two main *Dactylorhiza* clades, *D. fuchsii-maculata* and *D. incarnata-euxina,* respectively. The preferred models are boxed. MLE, marginal likelihood estimate. b) Cloudogram of 18,000 trees obtained for ten *Dactylorhiza* diploid species for which at least two accessions were available. Posterior probabilities higher than 0.9 are indicated for the relevant clades. More details are provided in the text. Eux = *D. euxina*, Fol = *D. foliosa*, Fuc = *D. fuchsii*, Ger = *D. gervasiana*, Ibe = *D. iberica*, Inc = *D. incarnata*, Sac = *D. saccifera*, Sam = *D. sambucina*, Umb = *D. umbrosa*, Vir = *D. viridis*. Photos: Sven Birkedal and Mikael Hedrén.

From the species delimitation analysis (Fig. 4a), the highest marginal likelihood estimate and best model for the *D. fuchsii-maculata* clade (including 14 individuals and 1837 independent SNPs) was the split model, distinguishing the four diploids: *D. fuchsii*, *D. foliosa*, *D. gervasiana*, and *D. saccifera*. The highest marginal likelihood and the best model for the *D. incarnata-euxina* clade (including 13 individuals and 2152 SNPs, each on a different RADtag) was recognition of *D. incarnata*, *D. umbrosa* (including “*D. osmanica*”), and *D. euxina* (Fig. 4a).

In the SNAPP species tree constructed for diploid *Dactylorhiza* (Fig. 4b), a similar overall topology was found as that recovered by the RAxML tree, with the exception of the subsequent placement of *D. iberica* and *D. sambucina* (Fig. 3); however, this relationship does not receive a high posterior probability in the species tree. Upon analyzing the SNAPP .log file in TRACER, all ESS values proved to exceed 200. The analysis of the SNAPP tree file with the TREE SET ANALYZER indicated that the 95% highest posterior densities (HPD) contained only four main tree topologies out of a total of 34 recovered. These four main topologies all placed *D. viridis* as sister to the rest of *Dactylorhiza*, but differed in the positions of *D. sambucina* and *D. iberica*; 50.5% of trees place *D. sambucina* as subsequent sister to the rest, including *D. iberica* as sister to all others, 23.4% of the trees show *D. sambucina* and *D. iberica* forming a clade that is sister to all remaining *Dactylorhiza* except *D. viridis*, and 18.1% of trees have *D. iberica* as sister to the rest, including *D. sambucina* as sister to all remaining *Dactylorhiza*. Finally, 3.2% of trees place *D. iberica* as sister to the *D. fuchsii-maculata* clade, whereas *D. sambucina* is shown as sister to the *D. incarnata-euxina* clade. The dated SNAPP tree indicates that divergence of the crown group of *Dactylorhiza* started in the late Miocene (Fig. 4b). The Mediterranean-Pontic *D. iberica* appears to have diverged around the period of the Messinian salinity crisis. However, the *D. fuchsii-maculata* clade seems to have split from the *D. incarnata-euxina* clade around the Miocene–Pliocene boundary, with most of the splits within each of these major clades inferred as having occurred within the last 2 myr, well within the Pleistocene.

**Figure 5.**
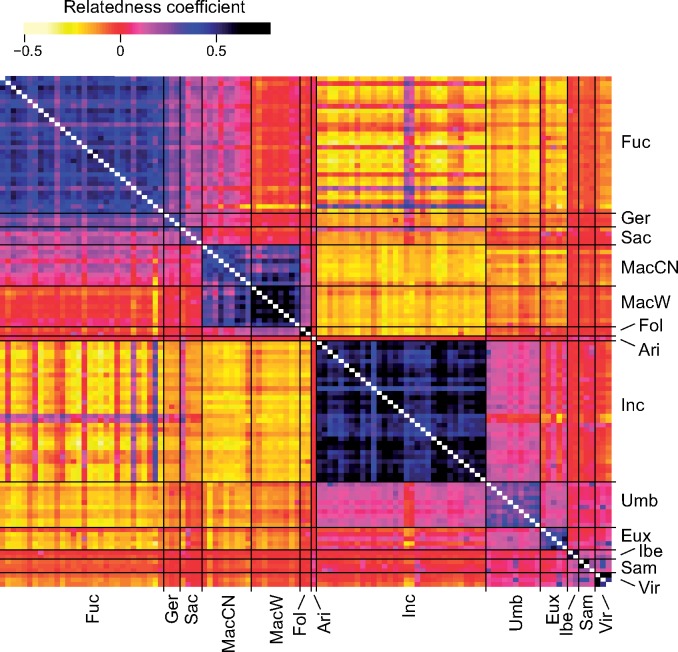
Heatmap of pairwise relatedness (Huang et al. 2014) between diploid and autotetraploid *Dactylorhiza* accessions. Ari = *D. aristata*, Eux = *D. euxina*, Fol = *D. foliosa*, Fuc = *D. fuchsii*, Ger = *D. gervasiana*, Ibe = *D. iberica*, Inc = *D. incarnata*, MacCN = central-northern *D. maculata*, MacW = western *D. maculata,* Sac = *D. saccifera*, Sam = *D. sambucina*, Umb = *D. umbrosa*, and Vir = *D. viridis*. To optimize color resolution, the estimates on the diagonal were excluded.

The relatedness heatmap (Fig. 5) complements well the RAxML results, pointing to a clear distinction between the *D. fuchsii-maculata* and *D. incarnata-euxina* clades. However, the heatmap shows several individuals of *D. fuchsii* potentially introgressed with *D. incarnata* alleles without a clear geographic pattern (results not shown); also some *D. incarnata* individuals show surprisingly high relatedness to *D. fuchsii* as an indication of recent gene flow. Further high between-species relatedness, viewed as signal for a relatively high gene flow, can also be identified, for example between *D. fuchsii* and *D. maculata* in Central Europe. The *D. incarnata-euxina* clade shares overall higher relatedness with *D. sambucina, D. iberica*, and *D. viridis* than with the *D. fuchsii-maculata* clade. In general, *D. viridis*, *D. iberica*, *D. incarnata*, *D. foliosa*, and W *D. maculata* show high within-group coancestry. In agreement, the highest relative per-individual inbreeding (Supplementary Fig. S2 available on Dryad) was found in $D$. *incarnata* and the Madeiran endemic *D. foliosa*, whereas the autotetraploid *D. maculata* and diploids *D. umbrosa*, *D. euxina, D. sambucina*, and *D. viridis* showed lower (but still mostly positive) values for inbreeding within the polymorphic positions retained in the vcf file.

### Allotetraploid Analyses

The coancestry heatmap based on ebg-derived polyploid genotypes (Fig. 5) generally clustered together the individuals of each of the 14 allotetraploid entities sampled for more than one individual. Among these taxa, *D. traunsteineri* appears to be the most heterogeneous, whereas *D. armeniaca* and *D. urvilleana* show the least within-group variability. Four main groups of allotetraploids can be distinguished that share more coancestry: (i) *D. urvilleana, D. pythagorae, D. armeniaca*, and (in part) *D. nieschalkiorum*; (ii) *D. sphagnicola, D. kerryensis, D. elata, D. brennensis*, and (in part) *D. nieschalkiorum*; (iii) *D. majalis, D. traunsteineri, D. purpurella, D. praetermissa, D. elatior,* and *D. baltica;* and (iv) *D. cordigera* and *D. kalopissii*. A highly similar pattern is observed in the phenetic network produced by SPLITSTREE (Supplementary Fig. S3 available on Dryad). This separates the *D. fuchsii*$\times$*D. incarnata* allotetraploids by visibly shorter distances than other allotetraploids. *Dactylorhiza traunsteineri* exhibits a weak geographical clustering between a northwestern group in Britain and western Norway, and a central-eastern European group.

**Figure 6.**
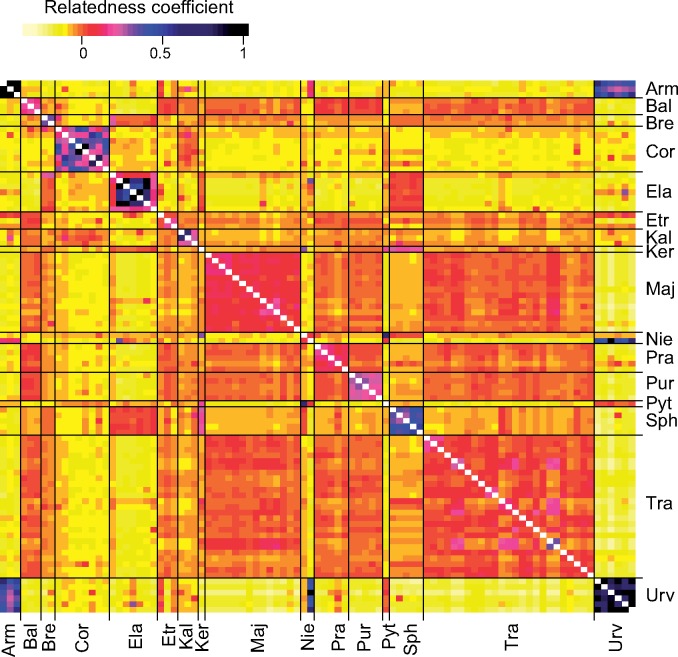
Heatmap of pairwise relatedness (Ritland 1996) between allotetraploid *Dactylorhiza* accessions. Arm = *D. armeniaca*, Bal = *D. baltica* (incl. *D. ruthei*), Bre = *D. brennensis,* Cor = *D. cordigera*, Ela = *D. elata*, Etr = *D. elatior*, Kal = *D. kalopissii* (incl. *D. macedonica*), Ker = *D. kerryensis*, Nie = *D. nieschalkiorum*, Maj = *D. majalis*, Pra = *D. praetermissa*, Pur = *D. purpurella*, Pyt = *D. pythagorae*, Sph = *D. sphagnicola* (incl. *D. calcifugiens*), Tra = *D. traunsteineri*, Urv = *D. urvilleana*. To optimize color resolution, the estimates on the diagonal were excluded.

The results of our relatedness analyses (Table 1, Fig. 6 and Supplementary Fig. S4 available on Dryad) indicate the following relationships between parental and derived clades:

(i) *Dactylorhiza fuchsii* and *D. incarnata* (or their ancestors) produced several central-NW European allotetraploids, including *D. baltica* (including *Dactylorhiza ruthei*; Supplementary Fig. S4e available on Dryad), *D. elatior* (Supplementary Fig. S4d available on Dryad), *D. majalis* (Fig. 6a), *D. praetermissa* (Supplementary Fig. S4c available on Dryad), *D. purpurella* (Supplementary Fig. S4b available on Dryad), and *D. traunsteineri* (Supplementary Fig. S4a available on Dryad). *Dactylorhiza fuchsii* was their maternal parent.
(ii) *Dactylorhiza maculata* (or its diploid ancestor, always as the maternal parent) and *D. incarnata* (or their ancestors) hybridized to form two NW European allotetraploids—*D. sphagnicola* (including *Dactylorhiza calcifugiens*; Fig. 6b) and *D. kerryensis* (Supplementary Fig. S4i available on Dryad)—and one SW-central European allotetraploid—*D. elata* (Supplementary Fig. S4j available on Dryad). For *D. elata,* the highest relatedness among the *D. incarnata-euxina* clade was in fact with *D. umbrosa* (Supplementary Fig. S6j available on Dryad). The difference between the relatedness estimators for *D. elata–D. incarnata* and *D. elata–D. umbrosa* was minute but nonetheless statistically significant. However, as several other relatedness estimators as calculated with POLYRELATEDNESS (results not shown) indicate *D. incarnata* to be closer than *D. umbrosa* to *D. elata*, and as only *D. incarnata* is currently sympatric with *D. elata*, we regard *D. incarnata* to be the more likely paternal parent of *D. elata*.
(iii) *Dactylorhiza saccifera* and *D. incarnata* (or their ancestors) were parents of the SE European *D. cordigera* (Supplementary Fig. S7d available on Dryad) and *D. kalopissii* (including *Dactylorhiza macedonica*; Supplementary Fig. S4g available on Dryad). *Dactylorhiza saccifera* was their maternal parent.
(iv) *Dactylorhiza saccifera,* as mother, hybridized also with *D. umbrosa* to form *D. pythagorae* (Supplementary Fig. S4h available on Dryad), another SE allotetraploid, endemic to Samos.
(v) *Dactylorhiza euxina* and *D. umbrosa* (or their ancestors) formed the endemic Turkish-Caucasian allotetraploid *D. armeniaca* (Supplementary Fig. S4f available on Dryad), for which *D. umbrosa* was the maternal parent.
(vi) Finally, *D. urvilleana*, with a distribution extending from northern Turkey to northern Iran, was produced by *D. euxina* and *D. saccifera* (or their ancestors; Fig. 6c), *D. saccifera* being the maternal parent.

**Table 1.** Details on the Dactylorhiza allotetraploids studied here

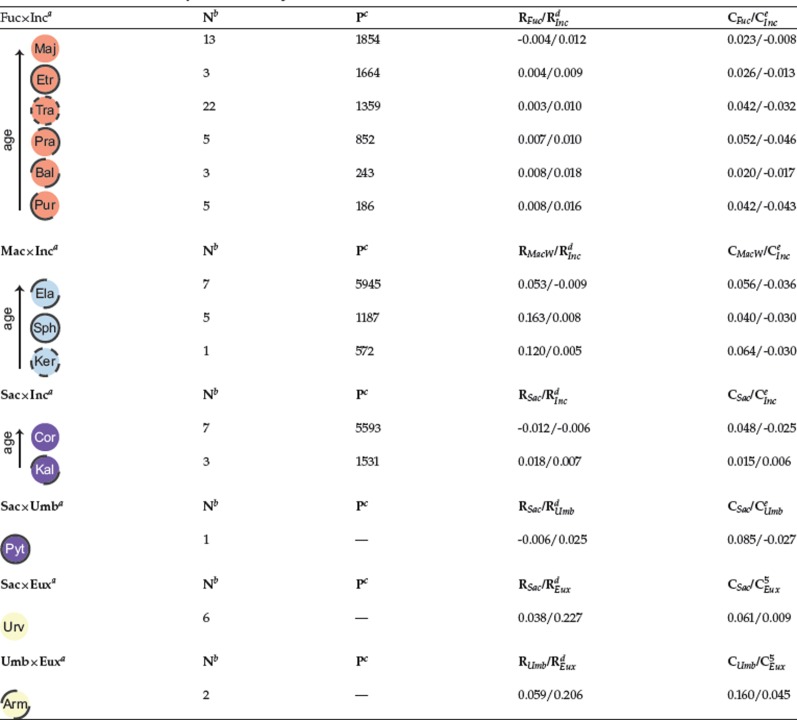

$^{a}$Arm = *D. armeniaca*; Bal = *D. baltica* (incl. *D. ruthei*); Cor = *D. cordigera*; Ela = *D. elata*; Etr = *D. elatior*; Kal = *D. kalopissii* (incl. *D. macedonica*) ; Ker = *D. kerryensis*; Maj = *D. majalis*; Pra = *D. praetermissa*; Pur = *D. purpurella*; Pyt = *D. pythagorae*; Sph = *D. sphagnicola* (incl. *D. calcifugiens*); Tra = *D. traunsteineri*; Urv = *D. urvilleana*. The parents involved in the origin of the allotetraploids are also indicated: Eux = *D. euxina*; Fuc = *D. fuchsii*; Inc = *D. incarnata*; Mac = *D. maculata*; Sac = *D. saccifera*; Umb = *D. umbrosa*.

$^{b}$Number of analyzed allotetraploid *Dactylorhiza* individuals.

$^{c}$Private alleles corrected for small sample sizes, calculated against the sibling allotetraploids (i.e., within the group of each parental pair).

$^{d}$Average relatedness estimates of each allotetraploid to each of its parents based on all genomic data.

$^{e}$Average relatedness estimates of each allotetraploid to each of its parents based on the RADseq loci that localized to the plastid genome.

Estimates of private alleles, corrected for small sample sizes for each allotetraploid relative to its sibling lineages (i.e., strictly within the same parental pair), range between 1854 and 186 for the *D. fuchsii*$\times$*D. incarnata* group, and between 5945 and 572 for the *D. maculata*$\times$*D. incarnata* group (Table 1). Within the larger family of *D. fuchsii*$\times$*D. incarnata* allotetraploids, the number of private alleles correlates significantly with the relatedness values to *D. fuchsii* ($P < 0.05$) but marginally fails the significance test to *D. incarnata* ($P$ = 0.06).

## Discussion

### Effectiveness of the RADseq Method for Resolving Reticulate Relationships

Despite the great frequency and evolutionary importance now attributed to hybridization and polyploidy (Linder and Rieseberg 2004; Mallet 2007; Giraud et al. 2008; Van der Peer et al. 2017; Taylor and Larson 2019), the subsequent restructuring of such polyploid genomes complicates phylogenetic inference. Several protocols have been recently proposed to process phylogenetic data that include polyploid and hybrid accessions (e.g., Gregg et al. 2017; Oxelman et al. 2017; Rothfels et al. 2017; Morales-Briones et al. 2018). Here, we take a phylogenomic approach to analyze a thoroughly sampled high-throughput sequencing data set for *Dactylorhiza*, arguably one of the most complex diploid–allotetraploid plant genera examined to date. There has been a long history of debate over the circumscription of both diploids and tetraploids, including and extensively beyond the taxonomic rank to which they should be assigned. These are common plants throughout their range, and determining how the plethora of morphologies and ecologies was formed has been a long-standing problem throughout Europe. Our approach to elucidating these issues included building a reference database of RADseq loci from diploids only and using multispecies coalescent to delimit and reconstruct relationships between the diploids within a dated species tree. We use these results to provide a framework for subsequent analyses of high-coverage sequence data for both diploid and tetraploid accessions. We applied a ploidy-aware calling algorithm and used estimators that are suitable for data sets including mixed ploidies.

The tens of thousands of variable positions derived from RADseq helped us to distinguish among 11 Eurasian *Dactylorhiza* diploids, 1 autopolyploid, and 16 allotetraploids, and to disentangle their putative parents. The RADseq phylogenetic trees of the diploids and autotetraploid *Dactylorhiza* species generally support prevailing taxonomies based on morphology and previous molecular studies with more limited taxon sampling and markers, but significantly improve upon phylogenetic resolution and thus estimated divergence times. Additionally, the RADseq matrix for the allotetraploids provides much new and refined data on recurrent allopolyploidization events in the genus, plus a deeper understanding of phylogeographic patterns and relative ages of these events. The present results are compared with those of former single gene/low-sampling density molecular studies in Supplementary Table S1 available on Dryad.

### Diploid Phylogenomic Analyses

The phylogenetic analyses performed on the putative parental Eurasian *Dactylorhiza* diploids identify several sequentially diverging species followed by two major sister clades (i.e., *D. fuchsii-maculata* and *D. incarnata-euxina*). The present phylogenetic trees confirm that *D. viridis* is the earliest diverging species within *Dactylorhiza* (Bateman et al. 2018a), splitting from the rest of *Dactylorhiza* less than 12 Ma. This age estimate is in general agreement with earlier estimates of a divergence time between *Dactylorhiza* and *Gymnadenia* in the second half of the Miocene (Inda et al. 2012). As in Brassac and Blattner (2015), the phylogenetic analysis in this study was performed both on a concatenated data set as well as treating each marker independently with a coalescent approach (Figs. 3 and 4). Both of these methods have their shortcomings and strengths. In the case of concatenated data sets, inconsistencies arise in the presence of incomplete lineage sorting and hybridization, sometimes artificially inflating statistical support for incorrect topologies as the data set grows larger (Degnan 2013; Liu et al. 2015). In the case of coalescent methods, the choice of markers, delimitation of species, and computational time are complicating factors (Liu et al. 2015; Fernandez-Mazuecos et al. 2017).

The European flora has experienced dramatic contraction–expansion phases associated with Pleistocene glaciation cycles (Hewitt 1996, 2000). Consequently, populations from northern Europe often exhibit lower levels of genetic diversity compared with those in the south that persisted locally throughout the cold periods (Taberlet et al. 1998). Within the *D. incarnata-euxina* clade, *D. umbrosa* and *D. euxina* are genetically variable and occur in the Near East and the Caucasus, pointing to a southeastern ancestral distribution of this group (Fig. 4). In contrast, European *D. incarnata* is genetically depauperate (Supplementary Fig. S3 available on Dryad), suggesting that it may have experienced strong genetic bottlenecks during its relatively recent (Fig. 4) expansion from Asia into Europe (Hedrén 2009). Alternatively, more recent losses of genetic diversity could have occurred, either in southern European refugia during the last ice age or during postglacial recolonization of northern Europe (Supplementary Fig. S3 available on Dryad; Balao et al. 2016, 2017). The restricted diversity in *D. incarnata* is reinforced by comparatively high levels of inbreeding and a patchy/localized distribution (Hedrén and Nordström 2009; Pedersen 2009; Naczk and Chybicki Zêtara 2016). The fact that *D. euxina* has hybridized with *D. umbrosa* to form an allotetraploid with disomic inheritance, *D. armeniaca* (Hedrén 2003), shows that these two diploid genomes are genomically well differentiated.

We estimated the split between the *D. fuchsii*/*D. gervasiana*/*D. saccifera* and *D. maculata*/*D. foliosa* clades to have occurred at approximately 4 Ma. *Dactylorhiza gervasiana* and *D. saccifera* have southern distributions, the former in the central Mediterranean area and the latter extending from the Hellenic Peninsula to the Caucasus. As these southern regions experienced relatively stable conditions throughout the Pleistocene glaciations, regional populations of these two species may have persisted in isolation for long periods. In spite of its wide distribution in central and northern Europe, *D. fuchsii* has relatively strong genetic cohesion across its distribution without geographic structure. These properties can be explained by a potentially rapid expansion from a southern refugium after the last ice age. *Dactylorhiza fuchsii* exhibits high levels of genetic diversity (Supplementary Fig. S3 available on Dryad), a pattern consistent with low inbreeding levels, a relatively even distribution, and comparatively efficient gene flow among populations across its wide distribution. Introgression from other species, in particular from *D. gervasiana* and *D. maculata*, appears to enhance within-species diversity of *D. fuchsii*.

*Dactylorhiza foliosa* is the only extant diploid member of the *D. maculata*/*D. foliosa* clade sampled here. It has a somewhat divergent morphology and is today restricted to the island of Madeira. It is possible that it colonized Madeira soon after emergence of the island at approximately 5 Ma, when the island was still linked to the Iberian peninsula by a chain of now submerged volcanic islands that could have acted as stepping stones for dispersal from the Eurasian continent (Geldmacher et al. 2000; Fernández-Palacios et al. 2011). The endemic nature of *D. foliosa* is reflected in its restricted genetic diversity (Supplementary Fig. S3 available on Dryad). Its relative, *D. maculata*, is invariably autotetraploid, but exhibits considerable geographically correlated variation. The western European population is genetically coherent and closely related to *D. foliosa* (Figs. 5 and Supplementary Fig. S4 available on Dryad), suggesting an exclusive common ancestor. In contrast, central and northern European accessions of *D. maculata* are more genetically divergent and could include tetraploids derived from hybridization and introgression with *D. fuchsii*, a process that may have been facilitated by the presence of some autotetraploid populations of *D. fuchsii* in Central European mountains, especially the Alps (Ståhlberg and Hedrén 2009, 2010). In contrast with *D. foliosa*, *D. maculata* displays higher genetic diversity (Fig. 5), as expected in an established autotetraploid with polysomal inheritance (Otto 2007). Due to the close relatedness of *D. foliosa* to western *D. maculata*, the allopolyploids formed with western *D. maculata* as parent also show high relatedness values with *D. foliosa*. However, we propose that either the widespread *D. maculata* or its (potentially extinct) mainland diploid ancestor is the more likely parent of western non-Madeiran allotetraploids such as *D. elata* (Supplementary Fig. S4 available on Dryad).

### Allopolyploid Evolution

Allopolyploidy is of fundamental importance for understanding angiosperm diversification (Stebbins 1980; De Bodt et al. 2005; Jiao et al. 2011; Van der Peer et al. 2017). However, few genomic studies have been performed in medium-aged polyploid complexes such as the *Dactylorhiza* complex studied here. Previous studies have focused on (very) young polyploid complexes to study the genomic effects of the allopolyploidization process (Song et al. 1995; Soltis et al. 2014) or to estimate numbers of polyploid origins (Chase et al. 2003; Soltis et al. 2004). However, such allopolyploids may not yet be established in natural habitats and should still contain two full genomes minimally affected by intergenomic recombination (Otto 2007). Other studies have been focused on ancient polyploid complexes with allopolyploids that have experienced millions of years of evolution, encompassing karyotype evolution, climatic oscillations, differential gene loss, functional gene diversification, and concerted evolution (Chase et al. 2003; Adams and Wendel 2005).

The age of the allotetraploids analyzed in this study may range from those that have evolved in previously glaciated areas after the last ice age, which we can link to their actual parental populations, to those that evolved long before the last ice age, for which recognizable parents are no longer extant (but nonetheless are closely related to the diploid descendants that we have successfully identified). The times of origins for most tetraploids appears to be associated with recent glacial cycles, as has been hypothesized for some other flowering plant genera (e.g., Novikova et al. 2018). Given that the allotetraploids analyzed here are typically more closely similar to either of the *D. fuchsii*/*D. gervasiana*/*D. saccifera* and *D. foliosa*/*D. maculata* clades and that these clades diverged from each other at about 4.5 Ma, none of the allotetraploids can be older than the split between these sister clades. Moreover, several allotetraploids revealed closer genetic similarity with either *D. saccifera* or *D. fuchsii* as one of their parents and *D. incarnata* or *D. umbrosa* as the other parent. As these closely related parental taxa in both cases diverged at less than 2 Ma, it is unlikely that any allotetraploid is older than this date. However, some allotetraploids are characterized by having common and geographically widespread plastid genomes that have not been identified in any extant member of the parental clades (Pillon et al. 2007; Nordström and Hedrén 2008, 2009). We found that such allotetraploids are also characterized by larger proportions of private alleles than those sharing plastid genomes with present-day members of the parental clades. Good examples of relatively old allopolyploids possessing many private alleles include *D. elata* in southwestern Europe and northwestern Africa and *D. cordigera* in southeastern Europe. Since these species are most closely related to parental clades in the same geographic areas, they probably originated in vicinity of the regions where they still occur today. These regions were less affected by climate changes during Pleistocene glaciations, so it is possible that *D. elata* and *D. cordigera* passed through multiple glaciation cycles.

In contrast, some of the studied allotetraploids exhibit low numbers of private alleles compared with their sibling allotetraploids, have high genetic similarity to particular parental species found within their distribution areas, and have their entire distributions confined to areas glaciated during last ice maximum. We conclude that such allotetraploids originated postglacially, a statement supported by findings that they share major allozyme alleles, plastid haplotypes, and ITS variants with their putative parents (e.g., Hedrén 1996; Devos et al. 2003; Pillon et al. 2007; Bateman 2011). Examples include *D. purpurella* (*D. fuchsii*$\times$*D. incarnata*) with an Atlantic distribution in northwestern Europe and *D. kerryensis* (*D. maculata*$\times$*D. incarnata*) endemic to Ireland.

The wide range of genetic similarities between allotetraploids and their putative parents suggests that allopolyploids may be of different ages and that the exact ancestors of some of them may be extinct or at least evolved significantly themselves after giving rise to the polyploids. For example, the genetically variable *D. elata*, confined to northwestern Africa and southwestern Europe, could have arisen from now extinct diploid members of the *D. maculata* clade that must have been present in the same general area and are still represented by the Madeiran endemic *D. foliosa*. In contrast, northwestern allotetraploids, including *D. sphagnicola* and *D. kerryensis*, are extremely closely related to the extant western European autotetraploid *D. maculata* (Fig. 6b and Supplementary Fig. S4 available on Dryad).

**Figure 7.**
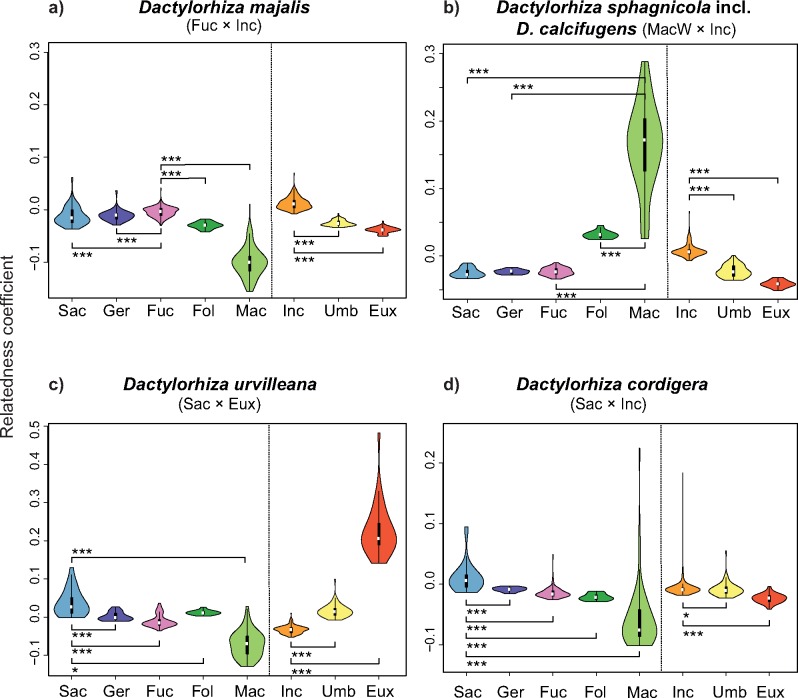
Examples of violin plots of relatedness of *Dactylorhiza* allotetraploids to potential ancestral genomes (diploid or autotetraploid): a) *D. majalis*; b) *D. sphagnicola* (including *D. calcifugens*); c) *D. urvilleana*; and d) *D. cordigera*. Within each panel, plots of relatedness of the allotetraploids to members of the *D. fuchsii-maculata* clade are shown to the left of a dashed vertical bar and members of the *D. incarnata-euxina* clade to the right. Stars indicate significantly different distributions (*$P \quad <$ 0.05; *** $P \quad <$ 0.001). Eux = *D. euxina*, Fol = *D. foliosa*, Fuc = *D. fuchsii*, Ger = *D. gervasiana*, Inc = *D. incarnata*, Mac = *D. maculata,* Sac = *D. saccifera*, Umb = *D. umbrosa*. Plots for the rest of the allotetraploids are given as Supplementary Fig. S4 available on Dryad.

On basis of one nrITS study (Devos et al. 2006), it was shown that the NW European *D. praetermissa* contains several ITS types, only one of which is found in an extant diploid, the southern *D. saccifera*. However, our results show that *D. praetermissa* has a greater overall similarity to *D. fuchsii* than to *D. saccifera* (Fig. 7), suggesting that *D. praetermissa* also has a postglacial origin within its present distribution. In agreement with this modified scenario, more recent studies show that the nrITS type considered potentially diagnostic of *D. saccifera* by Devos et al. (2006) is also present at low frequency in some northwestern European populations of *D. fuchsii* (Pillon et al. 2007; Pedersen and Hedrén 2010; Hedrén et al. 2011).

Young allopolyploids are not only restricted to previously glaciated areas. The southern *D. kalopissii* (*D. saccifera*$\times$*D. incarnata*) and *D. armeniaca* (*D. euxina*$\times$*D. umbrosa*) also exhibit high genetic similarity (Table 1; Supplementary Fig. S4 available on Dryad) and agree in plastid haplotype with present-day members of their diploid parental taxa (Hedrén et al. 2007).

### Hybridization Between Independently Formed Allotetraploids

Previous studies of the allotetraploid *D. brennensis*, which occupies a restricted area in central France, have revealed that it features highly divergent plastid genomes (Hedrén et al. 2012). These plastomes are not known from any present-day representatives of the diploid parents, but one of them is also identified in a regional population of *D. elata* in southern France and another one in *D. praetermissa* and *D. majalis*, in which it is widespread. Here, we confirmed the close relationship of *D. brennensis* to *D. elata* and a cluster including *D. majalis* and associated taxa (Fig. 7 and Supplementary Fig. S3 available on Dryad). These findings strongly suggest that *D. brennensis* originated by hybridization between distinct allotetraploids with independent origins, a conclusion also in agreement with ITS/ETS sequence data (Devos et al. 2006).

Similar hybridization events may also have taken place repeatedly between other independently derived allopolyploids in the past; this would explain both their high genetic diversity and the difficulty we experienced in matching some of the investigated allopolyploids to their exact parents. One such example is *D. majalis*, which exhibits plastid genomes from both *D. fuchsii* and *D. maculata* (or its allotetraploids *D. elata* and *D. sphagnicola*; Fig. 7), even incorporating some that do not match any yet found in extant parents (Nordström and Hedrén 2009). *Dactylorhiza majalis* and *D. traunsteineri* share to a large extent genetic markers in sympatry (Balao et al. 2016), which points to extensive gene flow and suggests that the divergence between these entities is largely related to selection for specific habitats (Paun et al. 2010, 2011). Furthermore, a scenario of hybridization and gene flow between these species is also in agreement with the finding that *D. traunsteineri* exhibits a geographically structured pattern of variation (not shown), populations from the European continent being somewhat differentiated from those in the NW (recognized taxonomically as *D. traunsteinerioides* by some authors; Bateman 2011). Such a pattern could have arisen as a consequence of hybridization with different but closely related tetraploid species in different parts of the total distribution area (e.g., Hedrén M, et al. 2018). Other possible causes for a geographic variation pattern could be isolation by distance, secondary hybridization and introgression from local representatives of the parental diploids, or taxonomic aggregation of independently formed allotetraploids in the same named species due to morphological/ecological convergence.

Given a complex of multiple allotetraploid derivatives of several parental combinations of different ages, some of them now evidently extinct (or at least unsampled thus far), and acknowledging widespread hybridization and gene flow between them, accumulation of genetic diversity is enhanced until the tetraploids exhibit as much or in some cases more genetic diversity than the parental diploids. Such a diverse polyploid complex has the potential to evolve and is likely to produce subtly divergent taxa that occupy a variety of habitats, some not occupied currently by the diploids.

## Conclusions

In this study, we have analyzed allotetraploids ranging from perhaps a few thousand years old to some that putatively evolved before the last glaciation. Taxa representing a substantial time-span offer a unique opportunity to investigate recurrent allopolyploidization, which might be especially important in the establishment of young polyploid species (Soltis and Soltis 1999; Mallet 2007). We find that crosses between the same parental diploids have produced multiple independent allotetraploids. Similar patterns of recurrent polyploidization have been found in other polyploid complexes, such as *Achillea* (Guo et al. 2013), *Asplenium* (Perrie et al. 2010), *Leucaena* (Govindarajulu et al. 2011), *Nicotiana* (Chase et al. 2003), and *Oryza* (Zou et al. 2015). Although certain features of genome evolution may follow common paths from repeated polyploidization events between the same parental combinations (Song et al. 1995; Soltis et al. 2009), stochasticity plays a major role in producing allopolyploid derivatives that differ in morphology and various traits of adaptive importance. The polyploid complex in *Dactylorhiza*, encompassing a dozen independently derived allotetraploids, provides an excellent example of this process. The amount of diversity and relative age of the allopolyploids correlates well with predictions based on the postglacial history of the European flora (Hewitt 1996, 2000; Taberlet et al. 1998). The least diverse and obviously youngest allotetraploids are found in the northwestern and northern parts of the distribution, which were covered by ice sheets during the last glaciation, whereas more diverse and obviously older allotetraploids are distributed further south in regions only indirectly affected by Pleistocene glaciations (Bateman 2011).

With increasing age, allotetraploid species will gradually acquire increased numbers of unique (i.e., private) alleles compared with their younger siblings, and also with their parents. Relatedness values will therefore decrease, providing a feature that can be used to provide a relative time frame for the origin of allotetraploid entities. Multiple possible causes could be put forward for this pattern: (i) accumulation of novel alleles within allopolyploids by mutation and genomic rearrangements; (ii) gradual change in the ancestral diploids by accumulation of mutations; (iii) hybridization with other independent allopolyploids with different evolutionary histories; and (iv) introgressive hybridization with diploids, parental, and other related lineages. These processes may occur partly at random, but may also be linked to the environmental selection pressures acting in the diverse habitats now occupied by the allotetraploids. We have here documented the diversity of processes and timescales that have contributed to the landscape complexity associated with this group of orchids in which polyploidy has played a major role. We have discovered that local adaptation is driving diversification of this group at both ploidies, generating both morphological and ecological parallelisms in independent parts of their distributions and divergence among closely related entities. Our work creates a solid foundation for future genomic studies that will elucidate how these factors have generated the diversity that for so long has confounded taxonomists and ecologists.

## Supplementary Material

Data available from the Dryad Digital Repository: http://dx.doi.org/10.5061/dryad.j01ph32.
